# Application of Dairy Proteins as Technological and Nutritional Improvers of Calcium-Supplemented Gluten-Free Bread

**DOI:** 10.3390/nu5114503

**Published:** 2013-11-14

**Authors:** Urszula Krupa-Kozak, Natalia Bączek, Cristina M. Rosell

**Affiliations:** 1 Department of Chemistry and Biodynamics of Food, Institute of Animal Reproduction and Food Research of the Polish Academy of Sciences, Tuwima St., 10, Olsztyn 10-748, Poland; E-Mail: n.baczek@pan.olsztyn.pl; 2 Cereal Group, Institute of Agrochemistry and Food Technology (IATA-CSIC), Av Agustin Escardino 7, Paterna, Valencia 46980, Spain; E-Mail: crosell@iata.csic.es

**Keywords:** dairy proteins, gluten-free bread, dough consistency, technological properties, nutritional value, celiac disease

## Abstract

Effect of dairy proteins on gluten-free dough behavior, and nutritional and technological properties of gluten-free bread was evaluated. Experimental doughs, containing dairy powders, showed low consistency. Obtained gluten-free breads were rich in proteins, and, regarding the energy value delivered by proteins, they could be considered as a source of proteins or high in proteins. Applied dairy proteins affected the technological properties of experimental breads causing a significant (*p* < 0.05) increase of the specific volume, crust darkening, and crumb lightness, depending on the dairy supplementation level, rather than the protein type. Dairy proteins incorporated at a 12% level, significantly (*p* < 0.05) decreased the hardness; nevertheless, the highest amount of proteins tested led to the opposite effect. These results indicate that milk proteins tested could be successfully added to gluten-free bread with beneficial effects on technological and nutritional properties.

## 1. Introduction

Celiac disease (CD) is a chronic immune-mediated intestinal disorder that develops in individuals having genetic predispositions with multiple contributing genes. The most important are HLA-DQ2 and HLA-DQ8, however, non-HLA genes also contribute to the development of CD. Approximately 1% of the worldwide population is suffering from CD, and, thus, this disorder is classified as one of the most common food intolerances [1,2]. CD is related to permanent intolerance to gluten, a storage protein found in wheat (gliadin), rye (secalins), barley (hordeins), and, probably, in some oat (avenins) cultivars. A great deal is known on the sequential pathophysiological events driving the intestinal inflammatory cascade [3,4,5] The immune response in CD involves the adaptive, as well as the innate, and is characterized by the presence of anti-gluten and anti-transglutaminase 2 antibodies, lymphocytic infiltration in the epithelial membrane and the lamina propria, and expression of multiple cytokines and other signaling proteins. The disease leads to inflammation, villous atrophy, and crypt hyperplasia in the small intestine. Mentioned factors can contribute to malabsorption of several nutrients (iron, folic acid, calcium, and fat-soluble vitamins) [6], general malnutrition, and reduced body mass index (BMI) [7]. Currently, strict and life-long adherence to a gluten-free diet (GFD) remains the only effective treatment for CD.

Generally, gluten-free formulas and baked products are poor in proteins [8]. In traditional baking industry proteins derived from plants (proteins of soya) and animal origin (milk proteins and egg albumins) are frequently used [9,10]. Milk proteins are highly functional ingredients characterized by a significant nutritional value. They swell in a high level and are able to build up a network [11,12]. Next to the functional benefits, gluten-free products with milk proteins are affluent in calcium and proteins, and, thus, enriched in essential amino acids like lysine, methionine and tryptophan [13]. Milk proteins can be successfully added to gluten-free products with beneficial effects on the technological properties. Caseinates are good emulsifiers and stabilize the batter; isolated and concentrated whey proteins can form gels; high temperature skim milk powder exhibits high water-binding capacity [10]. Whey proteins increased the specific volume and decreased bread crumb hardness over time, while sodium caseinate demonstrated the opposite effect [14]. On the contrary, the addition of both - whey proteins concentrate and sodium caseinate to short biscuit formulation, raised hardness and intensified surface brownness [15]. To improve the nutritional value of gluten-free products by the addition of milk proteins particular attention should be paid to the lactose content [16]. Celiac patients are often susceptible to secondary lactose intolerance due to alterations of lactase secretion resulted from the villous atrophy [6]. The addition of high protein/low lactose dairy powders combined with optimal amount of water resulted in gluten-free breads rich in proteins, with dark crust and white crumb, good acceptability scores in sensory tests, an increase in loaf volume, and a decrease in crust and crumb hardness [17]. 

There is a justified need to improve the nutritional value of gluten-free products. The present study is a continuation of previous trials on the enhancement of the quality and nutritional value of gluten-free bread [18], this time focused on the fortification in proteins. The aim of the study was to enrich a gluten-free formulation, supplemented with calcium citrate in low-lactose dairy proteins, to evaluate its mixing and pasting behavior and to analyze the technological properties, overall quality, and sensory characteristics of obtained gluten-free bread.

## 2. Experimental Section

### 2.1. Materials

Corn starch (Huici Leidan SA, Huarte, Spain), potato starch (EPSA, Valencia, Spain), pectin (E 440(i), ZPOW Pectowin, Jasło, Poland), calcium citrate (Sigma-Aldrich, St. Louis, MO, USA), commercial sunflower oil, dried yeast (Lessaffre, Valladolid, Spain), sugar, salt, and tap water were used as the basic ingredients. Calcium citrate was added in an amount to provide 0.6% of elementary calcium. The diary components used were: calcium caseinate (CAS; PZH SM Lacpol, Murowana Goślina, Poland), sodium caseinate (NAS; POCh, Gliwice, Poland), spray dried whey protein isolate (ISO; Carbery Ballineen, Ireland), and hydrolyzed whey proteins (OPT; Carbery Ballineen, Ireland). The amount of protein components was determined on the basis of nutrition and health claims made on foods in such a way that the final gluten-free product was either a source of protein or high in protein [19].

### 2.2. Characteristic of Dairy Powders

#### 2.2.1. Chemical Composition of Dairy Ingredients

The moisture, crude proteins (N × 6.25), and ash contents were evaluated using the standard methods [20,21,22]. The results presented are the mean values of at least two replicates.

#### 2.2.2. Physical and Functional Properties of Dairy Ingredients

Particle size distribution was determined using a Mastersizer 2000 Particle Size Analyzer with a wet dispersion unit Hydro 2000 S (Malvern Instrument Ltd, Malvern, England). Samples (1–2 g) were suspended in isopropanol. In order to keep the sample suspended and homogenized, it was recirculated continuously through the measurement zone. Particle size distribution was assessed using the mean particle volume (D50) in six replicates for each sample.

The measurement of color was performed by using a Minolta colorimeter (Chroma Meter CR-400/410, Konica Minolta, Japan), equipped with a granular attachment after standardization with a white calibration plate. The color was expressed in accordance with CIE-L*a*b* uniform color space (CIE-Lab). The parameters determined were lightness L* (L* = 0 [black] and L* = 100 [white]), a* (−a* = greenness and +a* = redness), and b* (−b* = blueness and +b* = yellowness). Values were the mean of nine replicates.

Water absorption index (WAI) and water solubility index (WSI) were determined according the method of Anderson *et al*. [23] at room temperature (RT) and after heating. Oil absorption capacity (OAC) was determined according to the method of Lin, Humbert, and Sosulski [24]. The values presented are the average of three measurements.

### 2.3. Mixolab^®^ Measurements

Mixing and pasting behavior of the protein enriched gluten-free dough were evaluated using Mixolab^®^ (Chopin, Tripette et Renaud, Paris, France) [25]. The Mixolab^®^ curves were recorded to evaluate the effect of different dairy powders and variable amount of them. Potato starch (8.4 g), pectin (2.1 g), calcium citrate (3.0 g), sugar (2.6 g), and salt (0.8 g) were added to corn starch (31.4 g). The investigated protein powders (6.3 or 12.6 g) were added substituting the appropriate amount of corn starch and 41.7 mL of water. For the assays, 90 g sample was placed into the Mixolab^®^ bowl and mixed (15 min/30 °C). Subsequently, the temperature was raised to 90 °C (increase of 4 °C/min) and at 90 °C was held for 8 min. In the cooling phase the temperature was reduced until 50 °C (decrease of 4 °C/min), and 50 °C was held for 2 min. The mixing speed during the entire assay was 80 rpm. The values obtained are the mean of two replicates. Parameters recorded included: initial pasting temperature where initial increase of consistency was detected, maximum torque during heating for potato (C3) and corn (C3′) starches, gelatinization rate for potato (β1) and corn (β2) starches, minimum dough torque during heating (C4), cooking stability rate (γ), final consistency after cooling until 50 °C (C5), and gelling rate (δ).

### 2.4. Breadmaking Process

Potato starch (16 g), pectin (4 g), and calcium citrate (5.8 g) were added to corn starch (60.3 g). The investigated protein powders (12 or 24 g) were added substituting the appropriate amount of corn starch. Subsequently, sugar (5 g), dried yeast (5 g), and salt (1.5 g) were dissolved separately in tap water (80 mL) and added to the dry ingredients together with oil (2.5 g). The batter was mixed for 12 min in a Brabender farinograph SEW (Brabender OHG, Duisburg, Germany). The resulting batter was proofed for 20 min in a proofing cabinet (35 °C/70% relative humidity). Then, the batter was divided into 200 g samples, placed in baking tins and proofed for another 20 min under the same conditions. The baking was carried out in convection oven at 200 °C for 25 min (Eurofours type 10AB20W2, Gommergnies, France). The obtained loaves were divided into two groups: the first was analyzed directly after cooling to room temperature (2 h); and the second was packed in polyethylene bags and stored 24 h at ambient temperature (22 ± 2 °C) for crumb structure analysis and sensory evaluation.

### 2.5. Characteristic of Gluten-Free Bread

#### 2.5.1. Chemical Composition and Nutritional Value

Bread moisture [20], proteins [21], ash [22], and fat [26] contents were determined following the standard methods. Total carbohydrates were determined by difference subtracting 100 g minus the sum of protein, ash, and fat expressed in g/100 g. The energy value was calculated by multiplying the amount of each macronutrient by the corresponding conversion factor (4, 9, and 4 for protein, fat, and carbohydrates, respectively) [27]. The percentage of energy delivered by protein was calculated.

#### 2.5.2. Technological Parameters

The bread loaf weight and volume (rapeseed displacement method) were determined. The specific volume and bake loss were calculated [18]. The height/width ratio of the central 10 mm slices was determined using Image J software (National Institutes of Health, Bethesda, MD, USA) [28]. The energy status of water in bread samples was measured as water activity (a_w_) using an Aqua Lab Series 3 (Decagon Devices Pullman, USA) at 22 °C. The crust and crumb color was analyzed as previously described using a Minolta colorimeter. Values were the mean of nine replicates.

#### 2.5.3. Crumb Texture Properties

Texture profile analysis (TPA test) of the 24-h stored bread crumbs was performed using Texture Analyzer TA-XT2i (Stable Micro Systems, Surrey, UK) equipped with a 30 kg load cell and 25 mm aluminum cylindrical probe. Three middle slices of each loaf were evaluated. A 10 mm thick bread slice underwent a double compression test up to 50% deformation of its original height at a crosshead speed of 1 mm/s and a 30 s gap between compressions. From the two-bite texture profile curve the following texture parameters were obtained: hardness, adhesiveness, springiness, cohesiveness, chewiness, and resilience.

### 2.6. Statistical Analysis

One-way analysis of variance (ANOVA) and Fisher’s least significant difference test (LSD) using Statistica 7.1 (StatSoft, Kraków, Poland) were applied to define significant differences (*p* < 0.05) between samples.

## 3. Results and Discussion

### 3.1. Chemical, Physical and Functional Characteristic of Dairy Powders

Detailed characteristics of selected dairy powders (NAS, CAS, ISO, OPT), including chemical composition, and physical and functional properties, are provided in order to evaluate the effect of each individual powder on the gluten-free batter and bread (Table 1). High concentration of proteins was confirmed for all dairy ingredients tested (Table 1). Total proteins content exceeded 85%, with distinguishing concentration in sodium caseinate, containing over 94% of proteins. According to the suppliers, all dairy ingredients tested were poor in fat (1.0%–1.5%) and lactose (0.5%–2%). Caseinates (CAS and NAS) were richer in mineral compounds (ash; near 4%) than whey proteins tested. 

The measurement of particle size showed that the mean size of whey proteins particles was significantly higher in comparison with particles of caseinates tested, and ISO showed the highest particle size (Table 1). The color parameters of dairy powders indicated that they were very light, white, or creamy powders, characterized by high L* value, above 91, and in the case of NAS, near 94. The negative value of parameter a* (below−2) indicated greenish color and the positive b* values described the yellowish color, being significantly higher for the whey proteins. 

The ability to bind and hold water without syneresis is critical in many foods, thus, hydration properties were determined in the dairy proteins. Results indicated that CAS followed by NAS showed the highest value of WAI at room temperature (Table 1), however, that trend was reversed when this parameter was determined after heating. Although caseins are relatively hydrophobic, they contain regions of high, medium, or low hydrophobicity [29] and they bind about 2 g water/g, which is typical of proteins [30]. Whey proteins in their native form exhibit little water-binding capacity [31]. Nevertheless, heat-denaturated whey proteins, although retaining most of their secondary structure, are linked together and can have a perceived hydration of over 10 g of water/g protein, compared with 0.2 g water/g protein for whey protein in their native globular state [29]. Processing has also a considerable impact on solubility of dairy proteins (Table 1). At room temperature, whey proteins tested were soluble in very high degree, however the heat treatment impaired the solubility of these proteins. WSI of ISO and OPT decreased by over 80% and 70%, respectively. Whey proteins are susceptible to denaturation at temperature higher that 70 °C. When heated, the tertiary structure of protein globules are destroyed, then unfolding of the protein molecules and new protein-protein interactions occur [32]. In case of both caseinates tested the opposite situation was observed. Here, after boiling the WSI values for CAS and NAS increased by more than 15%. Analyzing the OAC, significant differences were found between the dairy proteins tested (Table 1). The highest OAC was observed for CAS, followed by ISO, and OPT, whereas NAS has the lowest oil absorption capacity, probably due to its highest protein concentration. According to Kinsella [33], the mechanism of fat absorption has been attributed mostly to the physical entrapment of oil, but as well may be influenced by lipophilicity of the protein concentrate. In protein powder foods fat binding can be influenced by the size of powder particles [34], however in the dairy proteins tested such correlation was not observed.

### 3.2. Effect of Dairy Powders on the Gluten-Free Dough Characteristics

**Figure 1:**
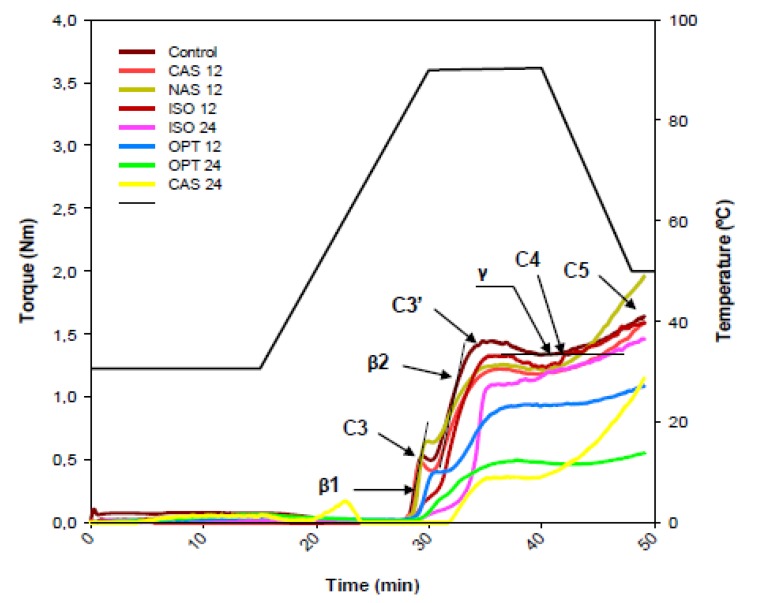
Effect of dairy proteins on the gluten-free dough consistency determined by Mixolab^®^ device. Control: unfortified gluten-free dough; CAS 12: gluten-free dough with 12% of calcium caseinate; CAS 24: gluten-free dough with 24% of calcium caseinate; NAS 12: gluten-free dough with 12% of sodium caseinate; NAS 24: gluten-free dough with 24% of sodium caseinate: OPT 12: gluten-free dough with 12% of whey proteins hydrolysate; OPT 24: gluten-free dough with 24% of whey proteins hydrolysate; ISO 12: gluten-free dough with 12% of whey proteins isolate; ISO 24: gluten-free dough with 24% of whey proteins isolate. C3: maximum torque during heating of potato starch; C3′: maximum torque during heating of corn starch; C4: minimum dough torque during heating; C5: final viscosity after cooling till 50 °C; β1: gelatinization rate for potato starch; β2: gelatinization rate for corn starch; γ: cooking stability rate.

The effect of the dairy powders at two different levels (12% and 24%) on the rheology of gluten-free dough was studied by using the Mixolab, where dough behavior subjected to shear and temperature constraints is recorded [25]. The Mixolab plots obtained in the presence of the dairy powders are shown in Figure 1.

Gluten-free doughs showed very low consistency during mixing, which only increased after heating when starches present in the recipe started to gelatinize. Consistency enhanced during heating till the rupture of the starch granules where the maximum consistency was detected. Further heating led to a decrease in the consistency derived from the starches stability during cooking and when temperature decreased the amylose retrogradation associated to cooling was observed as a consistency increase. This pattern agrees with previous description of the compounds changes reported by Rosell *et al*. [25]. The trends observed on the plots indicated that gluten-free dough rheology was clearly governed by starch changes during heating and cooling. The consistency of the gluten-free dough is greatly dependent on the amount of water or hydration, showing very low consistency during mixing when water adsorption is higher than 90% [35]. It must be remarked that during heating two consistency peaks were detected that were associated to the different gelatinization temperature of corn and potato starches. Matos and Rosell [36] also detected different peaks depending on the type of starch and their diverse pasting temperatures, being 65.4 °C for potato starch, 69.9 °C for corn starch and 70.2 °C for rice flour. In addition, Krupa-Kozak *et al*. [18] observed two different slopes during heating, the first one detected around 28–31 min corresponded to potato starch gelatinization, whereas the second one observed from 31 to 36 min was ascribed to corn starch gelatinization, which agrees with results of the present study. The patterns obtained during mixing, overmixing, pasting, and gelling greatly varied with the protein source and the level of proteins (Figure 1). It was not possible to record the consistency of the 24% NAS containing dough with the Mixolab due to its high consistency. The inclusion of the dairy proteins decreased the dough consistency during the heating-cooling stages, with the exception of NAS added at 12% that showed higher consistency after cooling. Bonet *et al*. [37] found that the addition of protein sources (gelatin, egg, and lupine) to wheat flour significantly changed the Mixolab plot and the effect was attributable to the nature of the proteins. The presence of different proteins and starches modifies protein–protein interactions and also the starch gelatinization and the gelling processes [25,35,38]. Regarding the level of the proteins added, dough consistencies decreased with increasing level of proteins. 

Primary and secondary parameters were extracted from the Mixolab curves to quantify the effect of the different dairy proteins on dough empirical rheology (Table 2). Proteins added to experimental doughs retarded the initial pasting temperature and the temperatures at which maximum dough consistency (C3 and C3′) was obtained; likely due to proteins compete with the starch for the available water, limiting the starch granule swelling and, therefore, promoting a delay in the pasting process as has been observed with hydrocolloids [39]. Consistency associated to potato starch gelatinization (C3) decreased in the presence of proteins, with the exception of NAS 12, and was barely noticeable when increasing protein level up to 24%. That effect was even more accentuated in the case of the consistency associated to corn starch gelatinization (C3′), since water will be even more limited after proteins hydration and potato starch gelatinization. Nevertheless, no relationship could be established between the consistency parameters during heating and the hydration properties of the dairy powders, likely the interaction between starches and proteins affected the ability of the proteins and starches to bind water. The final dough consistency after cooling (C5) was also significantly affected with the addition of the different protein powders, therefore, dairy powders modified amylose chains crystallization and in consequence, starch gelling, and the effect was dependent on the nature of the protein. NAS added at 12% increased the final consistency, whereas ISO at 12% did not modify that parameter and the other proteins decreased it. When increasing levels of proteins were added, the effect was a reduction of the final consistency. Considering the particle size of the proteins and also the hydration properties of the dairy proteins, there was no relationship between those properties and the dough behavior during heating and cooling. Regarding the secondary parameters, all doughs showed very low cooking stability range (C4–C3), whereas the cooling setback or gelling (C4–C5) was increased with the inclusion of dairy proteins, with the exception of the OPT that decreased that value. Likely the hydrolyzed nature of this protein isolate hindered the amylose recrystallization, lowering the final dough consistency. The rate of the starches phenomena associated to heating (β1, β2, γ) and cooling (δ) were significantly affected in the presence of proteins. In general, starch gelatinization was slowed down in the presence of proteins, with the exception of NAS-12 in the case of β1 or ISO-12 in the case of β2. Again, no correlation could be established with the protein properties. 

### 3.3. Chemical Composition and Nutritional Value of the Gluten-Free Breads Containing Different Dairy Powders

The addition of dairy proteins to gluten-free bread formulations is a common practice for increasing nutritional value, as in general, the commercial gluten-free formulas are poor in proteins [35,40]. Similarly to the results of our previous study [41], the control gluten-free bread, mainly composed of corn and potato starches, was poor in proteins (Table 3).

**Table 1:** Chemical, physical, and functional characteristic of dairy powders.

Sample	Moisture (%)	Protein ^1^ (% d.b.)	Fat ^2^ (% d.b.)	Lactose ^2^ (% d.b.)	Ash (% d.b.)	Particle size (µm)	Color parameters			WAI (g/g)		WSI (g/100 g)		OAC (g/g)
							L*	a*	b*	RT	90 °C	RT	90 °C	
CAS	7.23 ^a^	88.39 ^b^	1.50 ^a^	0.50 ^c^	3.99 ^a^	62.89 ^a^	91.78 ^b^	−2.09 ^a^	9.42 ^d^	2.81 ^a^	1.21 ^d^	56.84 ^b^	72.78 ^a^	1.85 ^a^
NAS	6.66 ^b^	94.42 ^a^	1.00 ^b^	1.00 ^b^	3.93 ^a^	75.79 ^b^	93.70 ^a^	−2.46 ^c^	6.77 ^c^	1.84 ^b^	1.54 ^c^	50.61 ^c^	67.95 ^b^	1.25 ^d^
OPT	4.43 ^c^	87.02 ^b^	1.00 ^b^	2.00 ^a^	2.69 ^b^	179.19 ^c^	91.37 ^c^	−2.48 ^c^	11.05 ^a^	0.96 ^c^	5.30 ^b^	81.88 ^a^	22.34 ^c^	1.37 ^c^
ISO	4.40 ^c^	88.42 ^b^	1.50 ^a^	0.20 ^d^	2.68 ^b^	233.70 ^d^	91.09 ^d^	−2.32 ^b^	10.86 ^b^	0.82 ^d^	6.12 ^a^	81.85 ^a^	14.17 ^d^	1.46 ^b^

CAS: calcium caseinate; NAS: sodium caseinate; OPT: whey proteins hydrolysate; ISO: whey proteins isolate; ^1^ N × 6.25; ^2^ Provided by the supplier; RT: Room temperature; WAI: Water absorption index; WSI: Water solubility index; OAC: Oil absorption capacity; Mean values labeled with different letters in the same column are significantly different (*p* < 0.05).

**Table 2:** Effect of dairy powders on the gluten-free dough characteristics during mixing and heating determined by Mixolab^®^ device.

Sample	Initial pasting temperature (°C)	Peak torque (potato) (C3) (Nm)	Temperature at C3 (°C)	Peak torque (corn) (C3′) (Nm)	Temperature at C3′ (°C)	β1 (potato) (Nm/min)	β2 (corn) (Nm/min)	C4 (Nm)	Γ (Nm/min)	C5 (Nm)	Cooking stability range (C4-C3) (Nm)	Gelling (C5-C4) (Nm)	δ (Nm/min)
Control	69.1 ^d^	0.53 ^b^	72.9 ^e^	1.45 ^a^	84.5 ^b^	0.618 ^a^	0.285 ^b^	1.34 ^a^	−0.026 ^b^	1.68 ^b^	−0.11 ^b^	0.35 ^c^	0.050 ^c^
CAS 12	71.2 ^c^	0.29 ^d^	74.1 ^d^	1.03 ^c^	86.2 ^a^	0.470 ^b^	0.227 ^c^	0.97 ^b^	−0.025 ^b^	1.45 ^c^	−0.07 ^ab^	0.49 ^b^	0.065 ^b^
NAS 12	72.4 ^ab^	0.66 ^a^	76.9 ^b^	1.25 ^b^	85.1 ^b^	0.726 ^a^	0.181 ^d^	1.21 ^a^	−0.012 ^a^	2.05 ^a^	−0.04 ^a^	0.84 ^a^	0.114 ^a^
OPT 12	73.3 ^a^	0.41 ^c^	78.3 ^a^	0.94 ^c^	86.3 ^a^	0.302 ^c^	0.149 ^e^	0.93 ^b^	−0.007 ^a^	1.12 ^d^	−0.01 ^a^	0.20 ^d^	0.032 ^d^
ISO 12	71.6 ^bc^	0.17 ^e^	75.3 ^c^	1.34 ^b^	86.6 ^a^	0.134 ^d^	0.360 ^a^	1.24 ^a^	−0.030 ^b^	1.70 ^b^	−0.11 ^b^	0.47 ^b^	0.039 ^cd^
Control	69.1 ^C^	0.53 ^A^	72.9 ^C^	1.45 ^A^	84.5 ^D^	0.618 ^A^	0.285 ^B^	1.34 ^A^	−0.026 ^C^	1.68 ^A^	−0.11 ^B^	0.35 ^B^	0.050
CAS 24	nd	nd	Nd	0.36 ^D^	85.1 ^C^	0.055 ^C^	0.131 ^C^	0.35 ^D^	−0.005 ^A^	1.28 ^C^	−0.02 ^A^	0.93 ^A^	0.117
NAS 24	na												
OPT 24	77.4 ^A^	0.21 ^B^	79.9 ^A^	0.50 ^C^	86.5 ^A^	0.096 ^B^	0.095 ^C^	0.47 ^C^	−0.008 ^B^	0.57 ^D^	−0.03 ^A^	0.11 ^C^	0.020
ISO 24	71.9 ^B^	0.09 ^C^	77.8 ^B^	1.11 ^B^	85.4 ^B^	0.047 ^C^	0.770 ^A^	1.09 ^B^	−0.008 ^B^	1.49 ^B^	−0.03 ^A^	0.40 ^B^	0.039

Control: unfortified gluten-free dough; CAS 12: gluten-free dough with 12% of calcium caseinate; CAS 24: gluten-free dough with 24% of calcium caseinate; NAS 12: gluten-free dough with 12% of sodium caseinate; NAS 24: gluten-free dough with 24% of sodium caseinate: OPT 12: gluten-free dough with 12% of whey proteins hydrolysate; OPT 24: gluten-free dough with 24% of whey proteins hydrolysate; ISO 12: gluten-free dough with 12% of whey proteins isolate; ISO 24: gluten-free dough with 24% of whey proteins isolate; C3: maximum torque during heating for potato starch; C3′: maximum torque during heating for corn starch; C4: minimum dough torque during heating; C5: final consistency after cooling till 50 °C; β1: gelatinization rate for potato starch; β2: gelatinization rate for corn starch; γ: cooking stability rate; δ: gelling rate; nd: not detected; na: not available; Mean values labeled with different letters in the same column are significantly different (*P* < 0.05). Lower case letters were used with the low-level protein addition and upper case letters are referred to the highest level of protein addition.

**Table 3:** Chemical composition and nutritional value of the gluten-free breads containing different dairy powders.

Sample	Moisture (%)	Proteins (% as is)	Fat (% as is)	Ash (% as is)	Energetic value (kcal as is)	Energy delivered by proteins (%)
Control	39.97 ^c ^	1.51 ^b ^	1.14 ^a^	1.82 ^d^	239 ^a^	2.53 ^e^
CAS 12	39.23 ^e^	8.65 ^a^	0.83 ^c^	2.14 ^a^	239 ^a^	14.50 ^b^
NAS 12	41.64 ^a^	8.57 ^a^	0.39 ^d^	1.82 ^d^	228 ^c^	15.03 ^a^
OPT 12	39.69 ^d^	8.23 ^a^	0.83 ^c^	1.98 ^b^	237 ^b^	13.86 ^c^
ISO 12	40.09 ^b^	8.07 ^a^	1.04 ^b^	1.92 ^c^	237 ^b^	13.61 ^d^
Control	39.97 ^C^	1.51 ^C^	1.14 ^D^	1.82 ^D^	239 ^C^	2.53 ^D^
CAS 24	40.59 ^A^	14.49 ^B^	1.29 ^C^	2.20 ^A^	235 ^D^	24.63 ^B^
NAS 24	39.37 ^D^	15.75 ^A^	1.21 ^CD^	2.15 ^B^	240 ^B^	26.25 ^A^
OPT 24	40.49 ^B^	14.74 ^B^	1.71 ^B^	2.06 ^C^	238 ^C^	24.74 ^B^
ISO 24	39.06 ^D^	14.39 ^B^	2.08 ^A^	2.12 ^B^	246 ^A^	23.43 ^C^

Control: unfortified gluten-free bread; CAS 12: gluten-free bread with 12% of calcium caseinate; CAS 24: gluten-free bread with 24% of calcium caseinate; NAS 12: gluten-free bread with 12% of sodium caseinate; NAS 24: gluten-free bread with 24% of sodium caseinate: OPT 12: gluten-free bread with 12% of whey proteins hydrolysate; OPT 24: gluten-free bread with 24% of whey proteins hydrolysate; ISO 12: gluten-free bread with 12% of whey proteins isolate; ISO 24: gluten-free bread with 24% of whey proteins isolate; Mean values labeled with different letters in the same column are significantly different (*p* < 0.05). Lower case letters were used with the low-level protein addition and upper case letters are referred to the highest level of protein addition.

As was expected, the proteins content in all experimental gluten-free breads with dairy powders increased significantly (*p* < 0.05). Breads supplemented with 12 g of milk powders were more than five-times richer in protein than the control. The increase of the level of supplementation with dairy proteins (up to 24 g) caused the further increase of protein content in the bread samples, especially in NAS 24 with sodium caseinate, which was ten-times richer in proteins than the control. Milk proteins have a high nutritional value [10,13] and the addition of milk proteins and essential amino acids, such as lysine, methionine and tryptophan also increases the nutritional value of the bakery products [14,15]. Within bread samples with dairy proteins addition, a fat content was low ranging from 0.39 to 2.08 (Table 3) as all dairy powders added were relatively poor in fat (Table 1). However, comparing with the control, experimental breads were more affluent in minerals, especially when 24 g of calcium caseinate was included to formulation (CAS 24). There are inconsistencies about CD patients being overweight at diagnosis and gaining weight while on GFD [42,43]. Taking this into account, the calorie content of gluten-free products is of importance [44]. Energetic value (in kcal) of experimental gluten-free bread with dairy proteins ranged from 228 to 246, and was comparable to unfortified control (239 kcal). Hager *et al*. [45] indicated that although the calorie content was higher in white and wholemeal wheat bread than in oat, buckwheat, maize, sorghum, teff, and rice, these breads were of inferior quality at the same time. In the present study the two levels of dairy proteins supplementation to experimental gluten-free bread were tested regarding the energy delivered by proteins (Table 3). In the case of experimental breads with lower amount (12 g) of caseinates and whey proteins powders, the proteins delivered around 15% or above 13% of energy, respectively. The higher threshold of proteins supplementation influenced further significant growth of energy delivered by proteins up to 26% in NAS 24. According to the European Parliament regulation on nutrition and health claims made on foods, a claim that a food is a source of protein may only be applied to food product where at least 12% of the energy value of the food is provided by protein, whereas a claim that a food is high in protein may only be made where at least 20% of the energy value of the food is provided by protein [19]. Based on that regulation, all experimental gluten-free breads with 12 g dairy proteins addition can be recognized as a source of proteins, whereas all experimental gluten-free breads with 24 g dairy proteins addition are high in protein.

### 3.4. Technological Parameters of Gluten-Free Breads with Dairy Powders

In general, milk products have been described as volume-depressing contributory factors of wheat bread [46,47]. In this study, the addition of 12 g dairy proteins to the experimental gluten-free formulations increased significantly the specific volume of all breads, comparing with the control bread, with distinguishing results obtained in bread NAS 12 (Table 4). Similarly, Gallagher *et al*. [17] indicated that addition of high protein/low lactose dairy powders resulted in gluten-free breads of improved overall shape and volume. Additionally, in the case of increase of sodium caseinate concentration in gluten-free formulation, the further increase of specific volume of bread NAS 24 was observed. In all remaining samples, the used of increased amount of dairy proteins (up to 24 g) affected specific volume in a different manner. Compared with the control, increased level of hydrolyzed whey proteins decreased significantly the volume of bread OPT 24, whereas a higher concentration of whey proteins isolate increased the specific value of bread ISO 24. Specific volume of bread CAS 24 was similar to the control. The volume of bread with dairy powders depends on the powder type and level of addition. Breads with addition of dairy supplements showed higher height/width ratio of central slices in comparison with the unfortified control (Table 4). However, only in the case of breads with the 12 g of proteins in formulation, the increase in the height/width ratio of slice was significant. Analyzing relationship between specific volume and H/W ratio a linear positively correlation (Pearson’s *r* = 0.49) was found. 

Applied dairy supplements, regardless of the amount, influenced the bake loss, defined as the amount of water and organic material lost during baking (Table 4). In comparison with the control, bake loss of all breads tested increased significantly. The opposite effect was observed only in bread OPT 24, where the value of bake loss was significantly reduced by higher concentration of hydrolyzed whey proteins. Crumb characteristic affects the rate of water transport [48]. Small size of crumb pore slowed down moisture migration [49], whereas a larger number of connections between gas cells would give a faster transport of water. Additionally, the number of connections of each gas cell increased with increased size of gas cell [50]. 

**Table 4:** Technological parameters of the gluten-free breads containing different dairy powders.

Sample	Specific volume (cm^3^/g)	Height/width ratio	Bake loss (%)	aw (av. temp.)	Crust color parameters			Crumb color parameters		
					L*	a*	b*	L*	a*	b*
Control	3.11 ^c^	0.80 ^d^	17.28 ^b^	0.981 (24.8) ^a^	46.37 ^a^	14.22 ^a^	35.34 ^a^	69.10 ^ab^	−1.25 ^b^	10.62 ^c^
CAS 12	4.50 ^a^	0.93 ^a^	22.00 ^a^	0.980 (24.8) ^a^	32.97 ^b^	11.75 ^b^	20.20 ^b^	66.02 ^c^	−1.01 ^a^	9.74 ^d^
NAS 12	4.70 ^a^	0.92 ^a^	22.74 ^a^	0.979 (24.9) ^b^	30.25 ^c^	8.31 ^e^	13.02 ^c^	69.92 ^a^	−0.95 ^a^	12.55 ^a^
OPT 12	4.56 ^a^	0.89 ^b^	22.42 ^a^	0.979 (25.0) ^b^	27.56 ^d^	9.76 ^c^	11.54 ^d^	68.79 ^b^	−1.18 ^b^	11.05 ^b^
ISO 12	4.00 ^b^	0.86 ^c^	19.90 ^a^^ b^	0.979 (24.8) ^b^	29.47 ^c^	9.23 ^d^	10.23 ^e^	68.95 ^ab^	−1.20 ^b^	9.80 ^d^
Control	3.11 ^C^	0.80	17.28 ^C^	0.981 (24.8) ^A^	46.37 ^A^	14.22 ^A^	35.34 ^A^	69.10^ C^	−1.25 ^E^	10.62 ^C^
CAS 24	3.10 ^C^	0.92	19.29 ^B^	0.979 (24.9) ^B^	29.34 ^B^	9.05 ^B^	10.65 ^B^	71.19 ^B^	−0.27 ^A^	14.55 ^A^
NAS 24	5.07 ^A^	0.86	22.13 ^A^	0.977 (25.1) ^D^	28.78 ^B^	5.34 ^E^	6.74 ^C^	65.09 ^D^	−0.53 ^B^	14.37 ^A^
OPT 24	2.43 ^D^	1.00	15.50 ^D^	0.976 (25.0) ^E^	29.12 ^B^	7.36 ^C^	6.62 ^C^	76.49 ^A^	−0.88 ^D^	14.81 ^A^
ISO 24	3.58 ^B^	0.85	19.11 ^B^	0.978 (24.8) ^C^	28.90 ^B^	6.61 ^D^	5.97 ^D^	69.92 ^C^	−0.80 ^C^	11.10 ^B^

Control: unfortified gluten-free bread; CAS 12: gluten-free bread with 12% of calcium caseinate; CAS 24: gluten-free bread with 24% of calcium caseinate; NAS 12: gluten-free bread with 12% of sodium caseinate; NAS 24: gluten-free bread with 24% of sodium caseinate: OPT 12: gluten-free bread with 12% of whey proteins hydrolysate; OPT 24: gluten-free bread with 24% of whey proteins hydrolysate; ISO 12: gluten-free bread with 12% of whey proteins isolate; ISO 24: gluten-free bread with 24% of whey proteins isolate; Mean values labeled with different letters in the same column are significantly different (*p* < 0.05). Lower case letters were used with the low-level protein addition and upper case letters are referred to the highest level of protein addition.

Incorporation of dairy powders affected the color of both, crust and crumb of experimental gluten-free breads (Table 4). Crust of the control bread was characterized by the highest lightness (L* = 46.37), whereas the inclusion of dairy proteins resulted in crust darkening, influenced by the level of dairy proteins addition rather than the protein type. All gluten-free breads containing dairy supplements showed the significantly lower L* value. Additionally, in the case of experimental gluten-free breads containing 12 g of dairy proteins, the further crust color diversification was observed. Crust of bread CAS 12 and NAS 12, containing calcium or sodium caseinate, respectively, was significantly lighter in comparison with dark crust of breads containing whey proteins, with distinguishing OPT12 where L* value reached 27.56. The value of parameter a* (red hue) was positive for crust of all experimental breads (Table 4). Comparing with the control, the incorporation of dairy powders to gluten-free formulation affected the significant decrease in redness, especially in breads containing sodium caseinate at both levels tested (NAS 12 and NAS 24). Crust of the control gluten-free bread obtained the highest b* value (yellow hue). Whereas, the addition of dairy proteins to formulation produced a significant decrease of crust yellowness of breads obtained, however all the values were still positive. The crust yellowness was especially low in the case of breads with higher dairy proteins concentration. Observed darkening of crust color resulted probably from the Maillard browning, a chemical reaction between amino groups and reducing sugars. In the case of milk derivatives undergoing a high temperature treatment, lactose as a reducing sugar interacts mainly with lysine residues, resulting in the formation of brown melanoidins [51]. These non-enzymatic reactions are responsible for numerous changes on food properties. From the technological point of view, the brown crust formation on gluten-free bread is desirable and the resulting color, taste and flavor characteristics are generally experienced as pleasant. Crumb color was influenced by a level of dairy proteins addition. Lower concentration of proteins tested in the formulations resulted in bread of similar to control crumb lightness, with slightly distinguishing NAS 12 (Table 4). Only in CAS 12 the L* was significantly reduced. Whereas, the lightness of bread crumb supplemented with 24 g of dairy proteins was higher in comparison with crumb of breads with 12 g dairy proteins addition, except for crumb of bread NAS 24. The a* values for the crumbs were all negative, with the lowest redness detected in the control crumb.

Comparing with the control, increasing concentration of dairy powders increased significantly the value of a* parameter of tested crumbs. This effect was especially visible in the case of breads containing calcium (CAS 24) and sodium (NAS 24) caseinate, where the a* value reached −0.27 and −0.53, respectively. Yellowness (b*) of all crumb samples was positive. Similarly to redness, also the value of b* increased significantly with increased dairy proteins concentration. Gluten-free breads containing dairy powders had an appealing dark crust and white crumb appearance, and received good acceptability scores in sensory tests [14,17]. As a wide variety of dairy supplement are available, their application in baked product development need to be determined adequately. In addition to the type and the amount of dairy supplement, the choice must be based on their physicochemical and functional properties, which varies remarkably.

### 3.5. Textural Parameters of Gluten-Free Breads with Dairy Powders

The values obtained for the textural parameters of the bread crumbs are shown in Table 5. Wide variations in the crumb hardness (3.66 to 25.28 N) were observed among the gluten-free bread samples. These results reflect large differences depending on type of proteins used. Dairy proteins incorporated at 12% level significantly (*p* < 0.05) decreased the hardness, with the exception of NAS 12. Nevertheless, the addition of increasing amounts of proteins led to the opposite effect and only ISO 24 remained softer than the control crumb. NAS at any level of addition led to harder crumbs and the same effect was observed in chewiness. Nunes *et al*. [14] analyzed the influence of low lactose dairy powders on gluten-free bread quality indicated that sodium caseinate had a negative impact on crumb hardness, whereas whey proteins demonstrated the ability to increase significantly the specific volume of the breads and decrease its the hardness.

**Table 5:** Texture profile analysis of the gluten-free bread crumbs containing different dairy powders.

Sample	Hardness (N)	Springiness	Cohesiveness	Chewiness (g)	Resilience
Control	9.44 ^b^	1.002 ^a^	0.454 ^ab^	431.70 ^b^	0.195 ^a^
CAS 12	3.66 ^e^	1.007 ^a^	0.460 ^a^	169.80 ^c^	0.186 ^a^
NAS 12	11.43 ^a^	0.981 ^ab^	0.427 ^b^	475.84 ^a^	0.188 ^a^
OPT 12	5.46 ^c^	0.940 ^c^	0.335 ^c^	170.74 ^c^	0.134 ^b^
ISO 12	4.20 ^d^	0.972 ^b^	0.366 ^c^	152.99 ^c^	0.141 ^b^
Control	9.44 ^C^	1.002 ^A^	0.454 ^B^	431.70 ^C^	0.195 ^A^
CAS 24	11.60 ^B^	0.979 ^AB^	0.486 ^A^	568.38 ^B^	0.196 ^A^
NAS 24	25.28 ^A^	0.954 ^B^	0.434 ^B^	1071.26 ^A^	0.184 ^A^
OPT 24	11.06 ^B^	0.872 ^C^	0.376 ^C^	368.83 ^D^	0.133 ^B^
ISO 24	6.35 ^D^	0.959 ^B^	0.446 ^B^	282.34 ^E^	0.181 ^A^

Control: unfortified gluten-free bread; CAS 12: gluten-free bread with 12% of calcium caseinate; CAS 24: gluten-free bread with 24% of calcium caseinate; NAS 12: gluten-free bread with 12% of sodium caseinate; NAS 24: gluten-free bread with 24% of sodium caseinate: OPT 12: gluten-free bread with 12% of whey proteins hydrolysate; OPT 24: gluten-free bread with 24% of whey proteins hydrolysate; ISO 12: gluten-free bread with 12% of whey proteins isolate; ISO 24: gluten-free bread with 24% of whey proteins isolate; Mean values labeled with different letters in the same column are significantly different (*p* < 0.05). Lower case letters were used with the low-level protein addition and upper case letters are referred to the highest level of protein addition.

Springiness is associated with a fresh, aerated and elastic product, thus high springiness is desirable in this type of products. Low springiness value is indicative of brittleness and this reflects the tendency of the bread to crumble when is sliced. Although the proteins addition decreased the springiness, the effect was only significant in the presence of the protein isolates (ISO and OPT) at both levels tested. Marco and Rosell [35] found springiness values that ranged from 0.77 to 0.94 when study the protein enrichment of rice based gluten-free breads, and later on Matos and Rosell [8] reported springiness values from 0.76 to 1.00 in commercial gluten free breads. Therefore, springiness values obtained in the present study agree with reported ones. 

Cohesiveness characterizes the extent to which a material can be deformed before it ruptures, reflecting the internal cohesion of the material. Bread with high cohesiveness is desirable because it forms a bolus rather than disintegrates during mastication, whereas low cohesiveness indicates increased susceptibility of the bread to fracture or crumble [8]. In case of breads containing caseinates (CAS and NAS) values obtained for cohesiveness were similar to control, while whey proteins (OPT and ISO) decreased, significantly, the value of this parameter (*p* < 0.05). Very low resilience values were obtained for experimental gluten-free breads, especially for breads with whey proteins, indicating a low elasticity. Values obtained agreed with results reported for commercial gluten-free bread where resilience ranged from 0.09 to 0.84 [8]. 

## 4. Conclusions

The present study has shown that the application of low-lactose dairy proteins in a gluten-free formulation influenced considerably the characteristic of experimental doughs and breads. Gluten-free doughs containing dairy proteins tested showed very low consistency during mixing stage and decreased consistency during the heating-cooling stages. Experimental breads were significantly richer in proteins and more affluent in minerals than the control one. Energetic value of experimental gluten-free bread with dairy proteins was comparable to unfortified control, however regarding the energy delivered by proteins they can be recognized as a source of proteins or as high in protein. Addition of dairy proteins to the experimental gluten-free formulations increased significantly (*p* < 0.05) the specific volume of all breads, with distinguishing results obtained in bread NAS 24. Inclusion of dairy proteins resulted in crust darkening and crumb lightness, influenced by the level of dairy proteins addition rather than the protein type. Dairy proteins incorporated at a 12% level significantly (*p* < 0.05) decreased the hardness, with the exception of NAS 12. Nevertheless, the addition of increasing amounts of proteins led to the opposite effect. Obtained results suggest that dairy proteins tested in this study could be used successfully in gluten-free recipes in order to obtain gluten-free bread of a pleasant color, taste, and flavor characteristics, and improved technological and nutritional properties. 
